# Best BLAST hit alone cannot be used as evidence of fraud

**DOI:** 10.1038/s41598-022-26720-y

**Published:** 2023-01-17

**Authors:** Natalia Díaz-Arce, Naiara Rodríguez-Ezpeleta

**Affiliations:** AZTI, Marine Research, Basque Research and Technology Alliance (BRTA), Txatxarramendi Ugartea Z/G, 48395 Sukarrieta, Bizkaia Spain

**Keywords:** Classification and taxonomy, Genetic markers

**arising from**: C. Blanco-Fernandez et al.; *Scientific Reports* 10.1038/s41598-021-91020-w (2021).

In a recent study, Blanco-Fernandez et al.^1^ applied molecular tools to authenticate fish products and conclude evidence of “worrying international fraud”. They revealed mislabeling in recognizable and unrecognizable fish products labeled as anchovy, hake and tuna commercialized by European companies. Their analyses consisted of extracting DNA from the fish product to be authenticated followed by amplification and sequencing of a suite of DNA markers and comparing the resulting sequences to the GenBank sequence database using BLAST (Basic Tool Alignment Search Tool) (https://blast.ncbi.nlm.nih.gov/Blast.cgi). By carefully reanalyzing their data, we identify errors in their identification of tuna species and conclude that best BLAST hit alone cannot be used as evidence of fraud.

Seafood product traceability is essential to detect intentional or unintentional mislabeling and thus helps reduce unreported, unregulated and illegal fishing while enhancing consumer safety^2^. Genetic methods have shown to be powerful for seafood product traceability^3^, in particular, when morphological characteristics cannot be confidently used such as in young age specimens (e.g., juveniles of bigeye and yellowfin tunas are very difficult to distinguish^4^) or in closely related species (e.g. black and white anglerfish^5^), and especially in processed products (e.g. filleted and canned), where anatomical traits important for fish identification (e.g. head, fins, skin) are absent. Accurate genetic based seafood product traceability requires developing approaches that unequivocally discriminate between species, for which it is essential to understand intra-specific variability as well as each species’ evolutionary context. In their study, Blanco-Fernandez, et al.^1^ use the best hit of a BLAST search against GenBank to assign species to the sample to be authenticated. Here, by analyzing their BLAST results considering other information, we show that using BLAST alone can lead to erroneous species assignments and thus to conclude fraud when there is not.

One of the fraud detections from Blanco-Fernandez et al. is the substitution of albacore (*Thunnus alalunga*) by Atlantic bluefin tuna (*Thunnus thynnus*), which is more than twice as expensive as albacore tuna (https://www.eumofa.eu/es/home). The authors explain this potential substitution by over-quota-caught bluefin tuna being sold as another species. This is a strong claim that needs clear evidence to be made. We thus examined the sequences corresponding to those albacore-labelled tuna products (MW557512, MW557513, MW557514) claimed to be mislabelled bluefin tuna due to a best BLAST hit with sequence EU562888, belonging to *T. thynnus* according to GenBank. Our hypothesis was that the mislabeling, rather than in the seafood products, is in the sequence in GenBank due to the mitochondrial introgression reported between *T. alalunga* and *T. thynnus*^6^. Indeed, it has been estimated that approximately 2–3% of *T. thynnus* individuals have the so-called “alalunga-like” mitochondrial DNA^7^, which has often misled mitochondrial based phylogenetic inferences of the genus *Thunnus*^8^. This hypothesis was confirmed by a phylogenetic inference including the putatively mislabelled sequences from Blanco-Fernandez, et al*.*^1^ and their best BLAST hit, as well as representative sequences from *T. alalunga*, *T. thynnus* (including those labelled as “alalunga-like”) and *T. albacares* (Fig. 1). The tree shows two clearly differentiated clades: one is exclusively composed by *T. thynnus* sequences, while the other includes *T. alalunga*, *T. thynnus* “alalunga-like” sequences as well as the sequences from the putatively mislabelled products and their best BLAST hit. These results refute the mislabeling of *T. thynnus* products as *T. alalunga* reported by Blanco-Fernandez et al*.*^1^.

**Figure 1:**
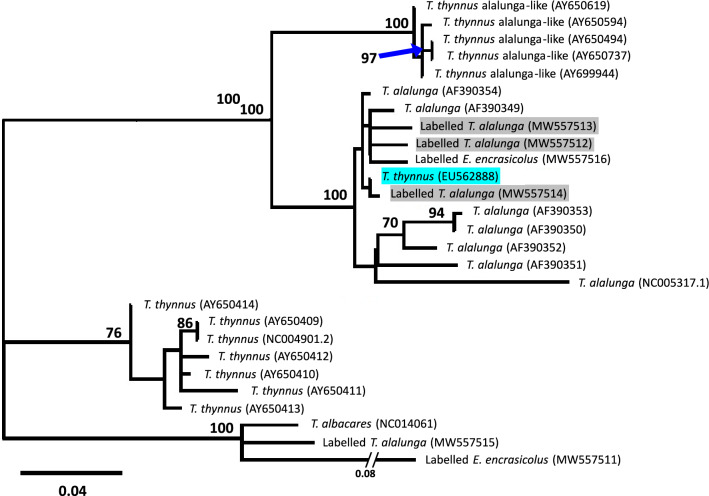
Maximum likelihood phylogenetic tree built using the GTRCAT model as implemented in RAxML version 8.1.21^9^ based on the mitochondrial control region (positions with less than 50% missing data between 5 to 414 of NC005317 sequence) aligned using Clustal W^10^ (default parameters) including all the available tuna identified sequences from Blanco-Fernandez et al*.* (MW557511-16) with the ones labelled *T. alalunga* matching to *T. thynnus* labelled in grey, the best BLAST hit *T. thynnus* sequence (EU562888), shaded in blue, and reference sequences from *T. albacares* (NC014061), *T. alalunga* (AF390349-54), *T. thynnus* (AY650409-14 and NC004901) and *T. thynnus* “alalunga-like” (AY650494, AY650594, AY650619, AY650737, AY699944). The tree is unrooted and branch support was assessed by a 100-replicate rapid-bootstrap analysis. Bootstrap values of those branches over or equal to 70% are indicated.

We have not found any other potential misidentification in Blanco-Fernandez et al*.*^1^, and therefore, their claim about existing fraud in highly appreciated fish still holds. Yet, this clear and easy to detect case in tunas could be the tip of the iceberg of errors made in food fraud studies relying solely on best BLAST hits to report mislabeling. Indeed, the bluefin tuna and albacore case is not an isolated one, and instances of genetic introgression that could lead to misidentification are increasingly reported in teleost fishes (e.g.^11,12^). Additionally, other factors such as traditionally used morphological characters for species assignment not being diagnostic can also occur and lead to false conclusions regarding mislabelling. This is the case of the black and white anglerfish for which mislabelling was reported^13^ based on the colour of their peritoneum as species diagnostic character, whereas it has recently been discovered that black anglerfish can have white peritoneum^14^ and, thus, reported mislabelling was most likely not so. As shown above, questioning our understanding of the evolutionary history of the species under investigation is essential for seafood fraud studies and could avoid errors such as the one made by Blanco-Fernandez et al. when they report mislabeling of albacore products.

Additionally, BLAST results deeply depend on the accuracy of the reference databases, and presence of contaminated sequences has been reported in GenBank^15^. Blanco-Fernandez et al.’s work increase the contaminations in GenBank as they have contributed sequences whose taxonomic assignment has relied on best BLAST hit. As consequence of this, there are now three sequences of *T. alalunga* in GenBank labelled *T. thynnus*. These sequences, as well as those not obtained from morphologically identifiable specimens, should be retracted from GenBank to avoid further ramifications.

In summary, we conclude that best BLAST hit cannot be used as evidence of fraud, and that studies on seafood authentication should consider the evolutionary context of the species under study. Not doing so can result in serious consequences as illustrated by the problems we found in the work of Blanco-Fernandez et al., who base their claims on tuna mislabeling trends in Spain on erroneous taxonomic assignments. Finally, besides using phylogenetic inference instead of BLAST search for genetic-based seafood authentication, we propose the generation of custom, tailored and curated reference sequence libraries specific for each case study that should be made publicly available. We recommend checking these reference libraries using phylogenetic quality control to detect misidentified or dubious sequences, and testing for adequate coverage for important species. In addition, we advise to review existing literature reporting known cases of interspecific hybridization or haplotype sharing involving included or closely related species. Finally, in order of avoid the inclusion of spurious sequences in public databases, we recommend the submission of sequence data produced only from identified species or to upload them as environmental samples otherwise.

## Data Availability

All data analysed for this reply have been downloaded from GenBank using the accession numbers provided in the figures.
